# A bibliometric analysis of current research, development status, and future development trends of soy and whey proteins

**DOI:** 10.3389/fnut.2025.1561792

**Published:** 2025-03-05

**Authors:** Meng-jie Li, Duo Feng, Meng-han Ma, Di Han, Hu Li, Na Li, Tian-xin Liu, Jia-yu Fan, Jing Wang

**Affiliations:** Institute of Food and Nutrition Development, Ministry of Agriculture and Rural Affairs, Beijing, China

**Keywords:** bibliometric, soy protein, whey protein, Citespace, dual protein

## Abstract

**Objective:**

This study aimed to analyse the current status and development of research on soy and whey proteins during the period 2004–2024 using bibliometric methods in order to reveal the research hotspots and development trends in this field.

**Methods:**

The study used CiteSpace and VOSviewer software to visualise and analyse 1,888 articles in the core database of Web of Science, including collaboration mapping, co-occurrence mapping, and co-citation mapping, in order to detect the development of disciplinary knowledge areas, research hotspots, frontiers, and trends.

**Results:**

The study found an overall fluctuating increase in the publication of relevant literature, especially a significant increase between 2016 and 2023. China has the most prominent research contribution in this field and maintains close collaboration with several countries. Keyword analysis revealed that research hotspots include antioxidant properties, sodium caseinate, pH effects, and colorectal cancer, and that research is gradually changing from process physicochemical properties to nutritional health. However, there were some limitations in this study, such as the narrow subject matter of the dataset, some high-quality literature may not be valued due to low citation rates, and there was a delay in updating the database, which affected the timeliness of the analysis.

**Conclusion:**

Over the past two decades, research on soy and whey proteins has witnessed a shift from processing aspects to nutrition and health, reflecting the emphasis and in-depth exploration of the nutritional and health values of proteins. Despite its limitations, this study provided a valuable reference for researchers in the field of soy and whey proteins, helping them to grasp the direction of their research and rationally allocate resources. At the same time, it also provided data support for policymakers to formulate relevant research policies and promote international cooperation, which will help to promote the further development and innovation of proteins in the field of nutrition. Therefore, it was recommended that research institutes strengthen their cooperation and work together to meet future research challenges and promote the sustainable development of proteins in nutrition.

## Introduction

With the improvement of living standards and health awareness, people are paying more and more attention to nutritional balance and health in their dietary choices. The rising concern for health and nutrition has also pushed the research on dietary protein nutrition to face great challenges and opportunities. Moreover, the continuous growth of the global population has resulted in a rising demand for protein resources. Amongst them, animal proteins, as an essential source of nutrition for the human body, tend to have a negative impact on the environment in the process of mass production. Therefore, strengthening diversified protein development and utilisation is an effective way of solving the problem of shortage of protein resources and realising sustainable development of the environment (1, 2).

*China National Nutrition Plan (2017–2030)* referred to the vigorous development of dual proteins, which were edible protein sources obtained from plant proteins, such as soy proteins, and natural high-quality proteins, represented by animal proteins, such as milk proteins, in accordance with the quantitative-efficacy relationship and precise interactions. Soy protein is the only high-quality plant protein that contains all essential amino acids and is rich in branched-chain amino acids, which can meet the human body’s demand for various essential amino acids (3). Studies have shown that soy protein is cholesterol-free and reduces the risk of cardiovascular disease, making it ideal for cardiovascular patients and vegetarians (4). In addition, soy protein contributed to blood sugar control, which was beneficial to the health of people with diabetes, and therefore also played an important role in the prevention and treatment of chronic diseases (5). Whey protein is a high-quality protein of animal origin with a variety of physiological functions, close to the human protein pattern, with a balanced amino acid composition, high biological value, and easy to digest and absorb (6). It plays an important role in promoting muscle growth and repair, as well as enhancing immune function, improving antioxidant capacity, and lowering blood cholesterol levels (7). Furthermore, whey protein could be used as a nutritional supplement to help restore strength in recovering patients, to support health maintenance in the elderly, and for fitness enhancement and recovery in athletes (8, 9).

In order to actively implement the national policy and deeply explore the functions of dual protein, it is necessary to use statistical methods to grasp the development trend and research hotspots of dual protein. Bibliometrics refers to the cross-science of quantitatively analysing all knowledge carriers by means of mathematics and statistics, integrating statistics, mathematics as well as vocabulary analysis, citation analysis, co-occurrence analysis, etc. It has become a key tool for exploring the trend of scientific publications by conducting in-depth profiling of academic literature in a specific field to reveal the interrelationships within the field of study and the academic influence of scholars and their teams (10, 11). The main aim of bibliometrics is to gain quantitative and qualitative insights into the structure, impact, and dynamics of the scholarly communication landscape. It helps researchers, institutions, and policymakers evaluate productivity, identify trends, and make informed decisions about research funding and collaborations, playing a crucial role in scientific and academic evaluation (12). In the context of the information age, the rise of interdisciplinary research has brought new development opportunities for bibliometrics, and its applied research in the fields of medicine, economy, environment, education, agriculture, food science and tourism, and so on continues to emerge (13–16).

There are a number of software tools proposed for the development of scientific mapping analyses (17–19). CiteSpace offers 11 features, collaborative mapping (author collaboration, country collaboration, and institutional collaboration) and co-occurrence mapping (feature words, keywords, and subject categories) for cited documents, and co-citation mapping (document co-citation, author co-citation, and journal co-citation) for cited documents (20). In addition, CiteSpace can detect the development of subject knowledge fields and their research hotspots, frontiers, and trends. By interpreting the mapping, it explains the current progress of the field and foresees the future development prospects of the field. VOSviewer is a literature visualisation and analysis software developed by Nees Jan van Eck and Ludo Waltman at The Centre for Science and Technology Studies (CWTS), Leiden University, The Netherlands (21). The software has a strong advantage in the analysis of authors, institutions, countries, and keyword co-occurrence, with clearer data presentation and more significant visualisation.

In this study, in view of the inaccuracy of the subject terms of dual protein in foreign journals, the subject terms were set as “soy protein/ soybean protein” and “whey protein,” and the visualisation software CiteSpace and VOSviewer were used to analyse the Web of Science (WOS) core database of the publication volume, authors, institutions, countries, journals, keywords, applied disciplines, and citation frequency of the literature were analysed in-depth. Multiple dimensions were analysed in-depth, and visual network maps were drawn. Meanwhile, this study analysed the research field of soy and whey proteins and the development trend of research hotspots in this field, to predict the future development trend and to stimulate the development and innovation of dual proteins.

The term “whey soy protein” (WSP) is an acid-soluble protein when the acid is adjusted to the isoelectric point of pH4.15 ~ 4.18 in the process of pumping soybean protein with water. This made it possible to include WSP in the literature search, so WSP-related contents are not specifically discussed in the analyses.

## Literature sources

**Figure 1:**
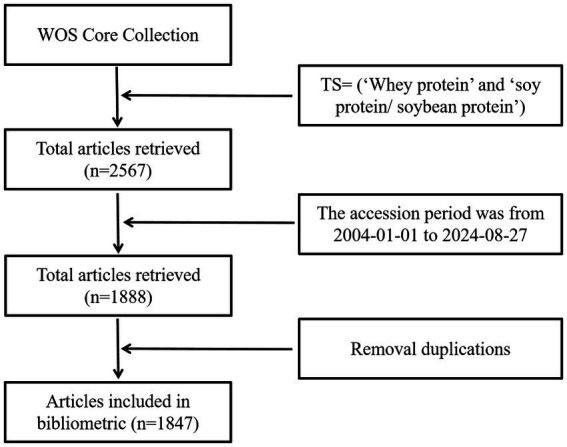
Articles’ search flowchart.

The core set of the WOS database was used as the English source database, and the search formula was TS = (‘Whey protein’ and ‘soy protein/ soybean protein’). The accession period was from 1 January 2004 to 27 August 2024, and the article types included articles and review articles, a total of 1,888 papers were obtained, and after eliminating duplicates, a total of 1,847 papers were included as the dataset of this study. The process for the selection and inclusion in the title catalogue is illustrated in Figure 1.

## Research method

The dataset was analysed and visualised using the bibliometric visualisation software CiteSpace 6.3.R1 and Excel 2019. The nodes in the CiteSpace visualisation map represent “author,” “institution,” “country,” “keyword,” etc. The size of the nodes reflects the number of related documents, and the larger the number of documents, the larger the size of the nodes. The strength of the connection between nodes and the thickness of the lines indicates the degree of association between them, which can reveal the interrelationships and cooperation networks amongst the topics in the research field.

## Results and analyses

### Results of the quantitative analysis of the volume of published literature

**Figure 2:**
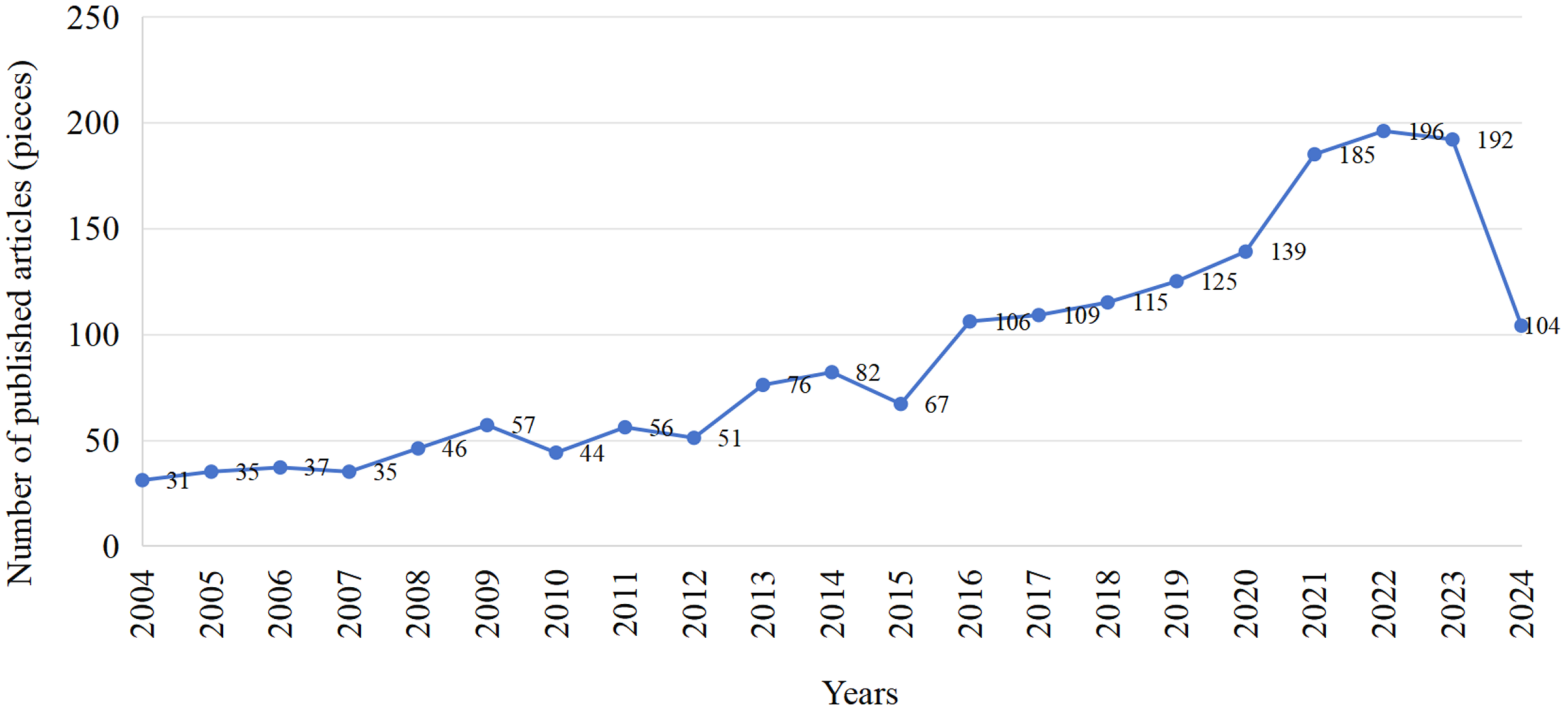
Annual publications of soy protein and whey protein-related studies in the Web of Science database from 1 January 2004 to 27 August 2024.

Since 2004, the number of articles published on the topic of soy and whey proteins has experienced fluctuating growth. As can be seen in Figure 2, the average number of articles published from 2004 to 2014 was 50, the average number of articles published in 2015 was lower, with only 67 articles, the average number of articles published from 2016 to 2020 was 119 and the average number of articles published from 2021 to 2023 was 191 and due to the limitation of the search time, not all the articles published in 2024 were included.

The trend of the line graph shows that the research in this field has been relatively stable between 2004 and 2010, with the research activity remaining at a relatively stable level. From 2011 onwards, the number of published articles started to show an upward trend, indicating that the field is gradually receiving more attention and research investment has increased, the number of published articles increased dramatically from 2016 to 2022, reflecting the rapid development of the field, which is very likely to have achieved a series of major research breakthroughs, thus attracting more researchers to participate in the field.

As shown in Figure 2, it clearly demonstrates the dynamic process of the number of relevant articles published between 2004 and 2024. Through an in-depth analysis of the trend at each stage, it is possible to gain precise insight into the research activity and development of the field at different times, as well as the possible influence of various factors. This is an important reference for researchers to grasp the direction of research, allocate research resources in a scientific and rational manner, and assess the development prospects of the field.

### Journals and co-cited journals

The 1,888 literature related to soy protein and whey protein was published in 461 journals, and as can be seen, the Food Hydrocolloids journal had the highest number of outputs (192 or 10.169%) in Table 1.

**Table 1:** Top 15 journals for published literature on soy protein- and whey protein-related research.

Publication titles	Record Count	% of 1,888	5 years IF
Food Hydrocolloids	192	10.169	11.3
Food Chemistry	92	4.873	4.1
LWT Food Science and Technology	90	4.767	3.7
Food Research International	62	3.284	7.4
Foods	61	3.231	5.1
Journal of Agricultural and Food Chemistry	58	3.072	6
Journal of Food Science	51	2.701	4.1
Journal of Food Engineering	50	2.648	5.3
International Journal of Biological Macromolecules	38	2.013	7.7
Nutrients	36	1.907	5.8
Journal of Nutrition	33	1.748	4.3
International Journal of Food Science and Technology	27	1.43	3.1
Journal of the Science of Food and Agriculture	27	1.43	4
Food Function	26	1.377	5.6
Food Bioscience	19	1.006	5.1

**Figure 3:**
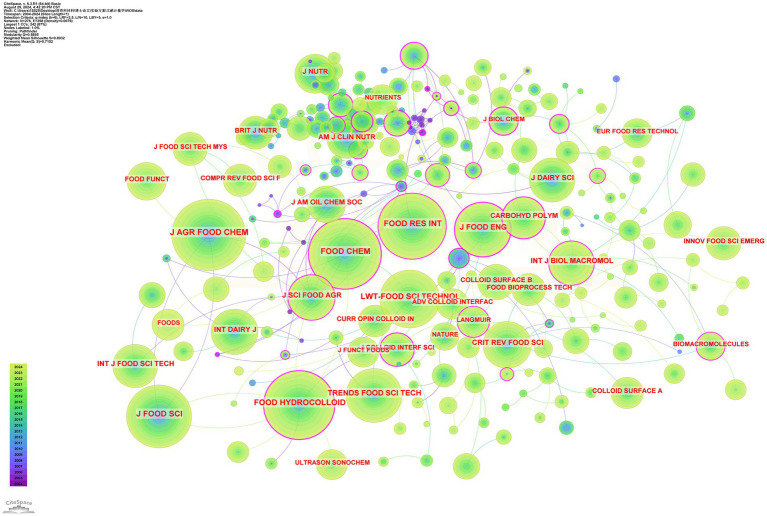
Visualisation of journal citation relationships for research related to soy protein and whey protein.

The cited journals were visualised by CiteSpace, where the node area represents how often the journal was cited, and the larger the node, the more influential the journal was in the field of study, and vice versa. Of the 24,682 co-cited journals, the results in Figure 3 show that the most highly cited journal was the Journal of Agricultural and Food Chemistry (1,296 citations), and the highest impact factor was food hydrocolloid (IF = 11.3).

### Author collaboration analysis

The data of 1,847 papers were imported into CiteSpace 6.3.R1 and VOSviewer software for processing, involving a total of 6,671 authors, and the role of high productivity had been instrumental in the development of this field of research. The top 5 authors were Jiang Lianzhou (21 articles), Mcclements David Julian (16 articles), Wang Xibo (10 articles), Wang Jing (9 articles), and Gao Yanxiang (9 articles).

Jiang Lianzhou and Mcclements David Julian focussed on the area of structural and functional properties of soy and whey proteins. Unlike Mcclements David Julian (22–26) focussed on the application of food hydrocolloids as well as food emulsifiers, whilst Jiang (27–31) focussed more on the flexible processing of proteins to achieve targeted modulation of functional properties. Wang Xibo’s research focussed on the properties of soy and whey proteins, and the improvement of their physicochemical properties after blending, especially in terms of the effect of ultrasonic treatment (32–36).Wang Jing focussed on the nutritional and health benefits of soy protein and whey protein and was committed to developing and creating new nutritious and healthy dual-protein foods (37–41).

**Figure 4:**
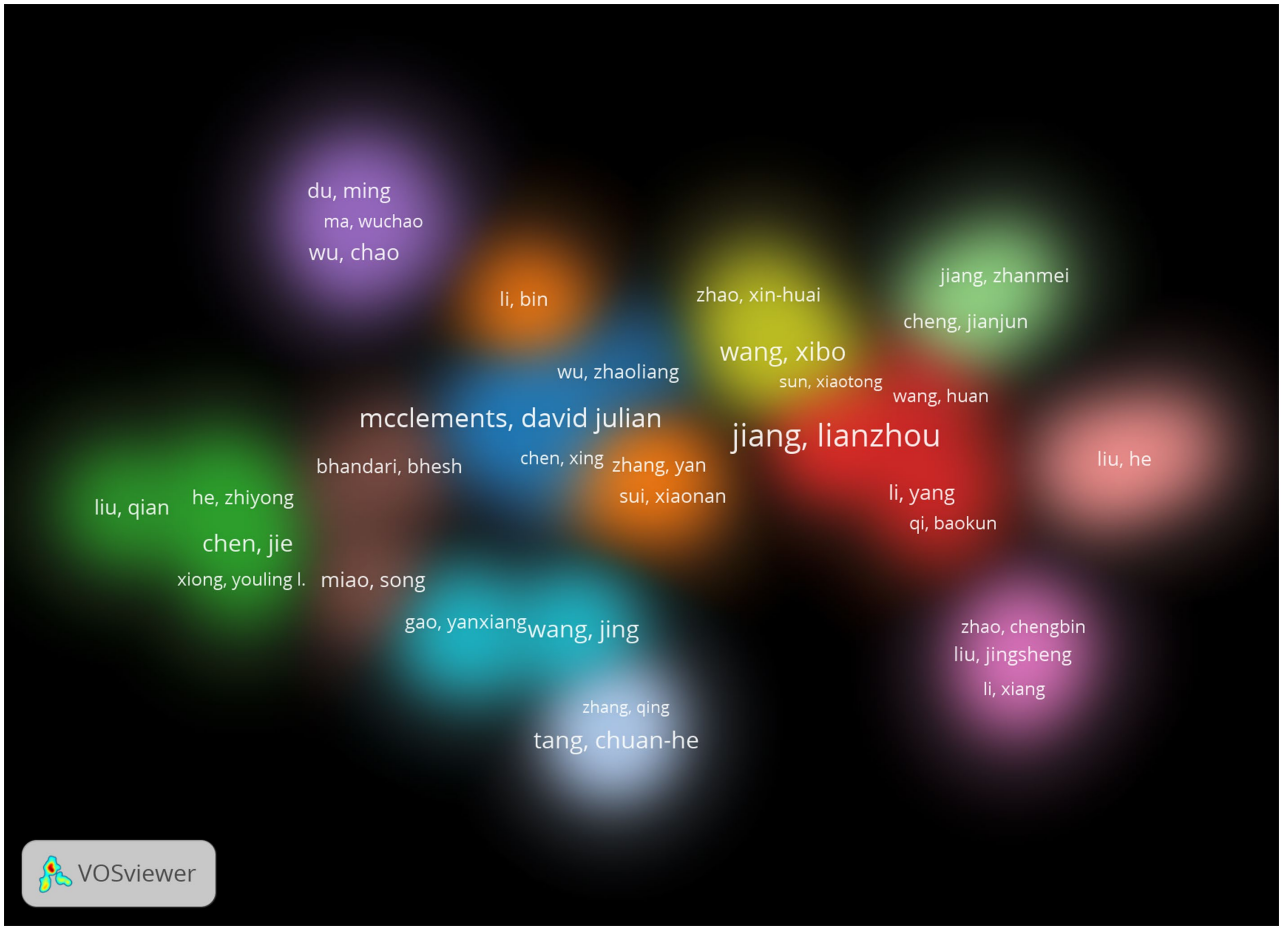
Density view of author collaborations in the literature related to soy and whey proteins.

The authors of 1,847 documents were screened to generate a view of the density of authors’ collaborative relationships (Figure 4), which resulted in the formation of 13 more closely linked research teams, with authors such as Jiang Lianzhou, Mcclements David Julian, and Wang Jing as the main representatives. This figure showed that the nodes are loosely distributed, indicating that although there are many researchers in this field, it has not yet formed a cluster of researchers of a certain size, with different research directions and a lack of inter-team cooperation and communication.

### Analysis of country/regional cooperation

**Figure 5:**
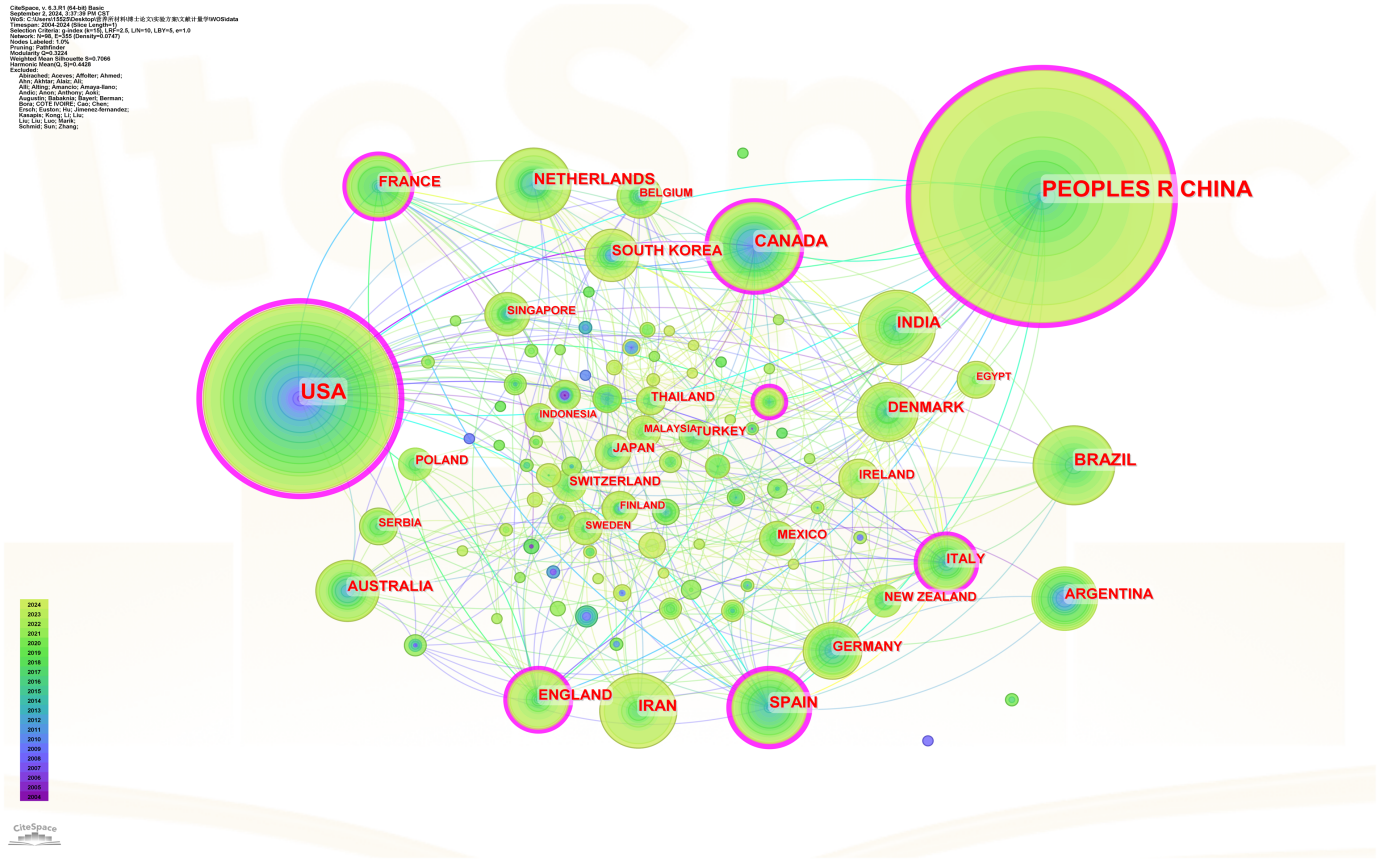
Analysis of national/regional collaborative networks in the field of soya and whey protein research. (Node size represents the number of publications, and different colours represent years.) The mapping shows that China is one of the core research forces in the field of soy protein and whey protein research with outstanding contributions and leading publication volume. The United States not only has a large number of publications but also works closely with many countries and has a significant influence on the international cooperation network. The Netherlands, Canada, and South Korea also have certain research outputs and actively carry out international cooperation. The overall network structure shows that research in this field is global, international, and diversified, and countries share resources and knowledge through cooperation to promote its development.

The 1,847 publications involved 96 countries, and amongst all the countries and regions involved in the research, China had the highest number of publications, followed by the United States, Canada, Brazil, the Netherlands, Spain, etc. The co-occurrence network diagram (Figure 5) demonstrated the strong connection between China’s research on soybean protein and whey protein and other countries and regions. The 10 countries with the highest number of articles are China (650 articles), the United States (365 articles), Canada (112 articles), Brazil (103 articles), the Netherlands (77 articles), Spain (76 articles), India (72 articles), Iran (66 articles), Australia (69 articles), and Argentina (53 articles). The level of activity in the research area in different countries and regions may be influenced by national policies and research funding support. For example, the *China National Nutrition Plan (2017–2030)* released by China mentioned the use of high-quality animal and plant proteins as the main nutritional base and focussed on the development of new nutritious and healthy foods such as dual-protein foods. The *Dietary Guidelines for Americans 2020–2025* suggested a healthy dietary pattern that recommends the intake of high-quality protein foods, such as soybeans and their products (42). Health Canada had released an assessment of the cholesterol-lowering health claims for soy protein (43). The *Dutch Dietary Guidelines 2015* for the population state that a shift towards a more plant-based and less animal-based diet improved health. They also highlight that the consumption of dairy products and milk is associated with a lower risk of colorectal cancer, while the consumption of legumes (including soybeans, lentils, chickpeas, and peas) helps reduce cholesterol (44).

### Analysis of institutional cooperation

**Figure 6:**
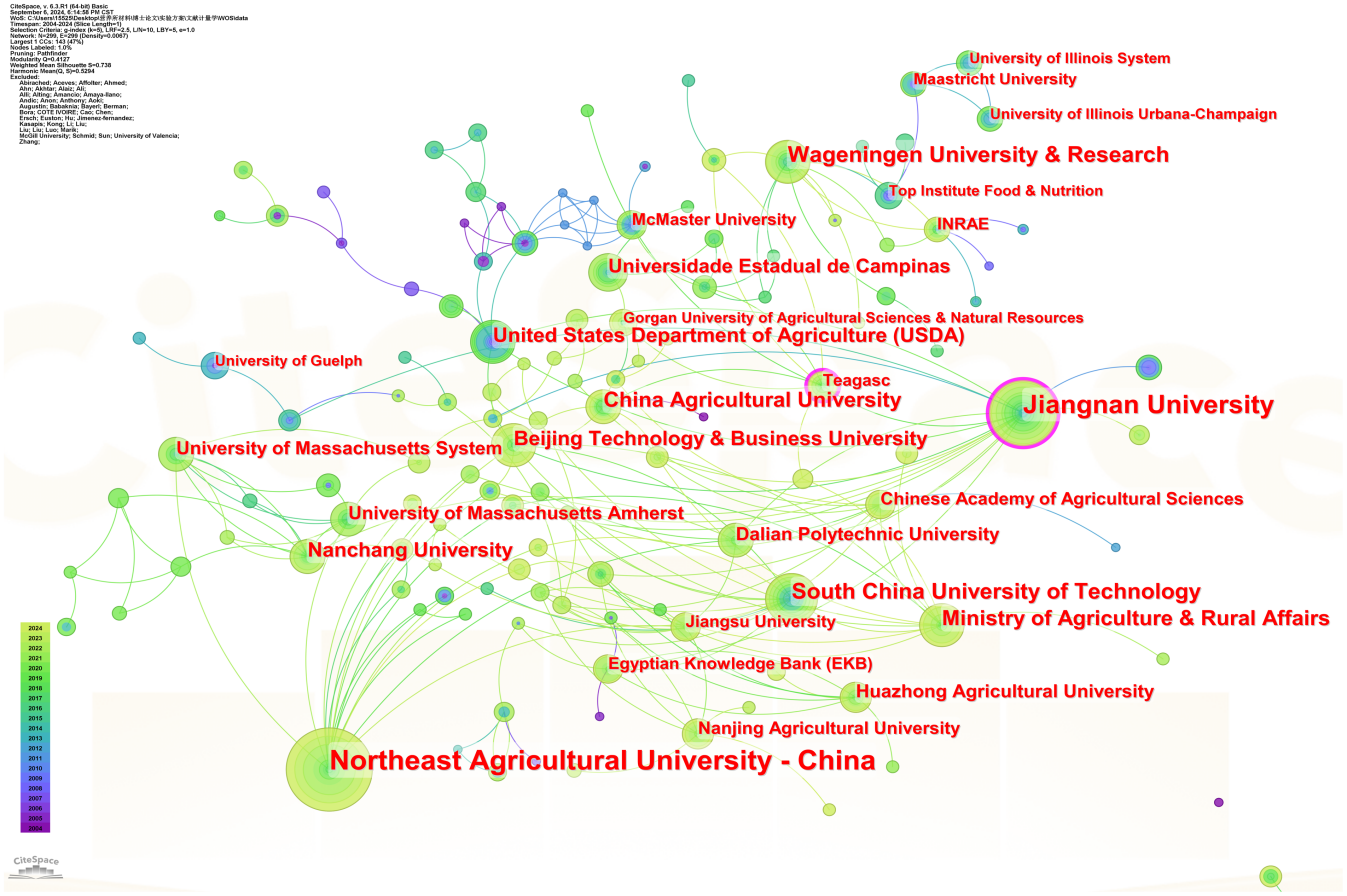
Collaborative network of research institutes. (Node size represents the number of articles published by the organisation, with larger nodes indicating a higher total number of published articles).

The institutional cooperation analysis was carried out by CiteSpace software, and the English literature was set to have a time slice of 1, yielding 299 nodes (Figure 6) (45). In the WOS core database, soy protein- and whey protein-related articles were mainly concentrated in universities, of which the top 10 are Northeast Agricultural University (104 articles), Jiangnan University (68 articles), South China University of Technology (38 articles), Consejo Nacional de Investigaciones Científicas y Técnicas (37 articles), Wageningen University (34 articles), Institute of Food and Nutritional Development of the Ministry of Agriculture and Rural Affairs (31 articles), China Agricultural University (30 articles), Nanchang University (28 articles), Beijing Technology and Business University (24 articles), and the United States Department of Agriculture (24 articles).

### Keyword analysis

#### Keywords

**Figure 7:**
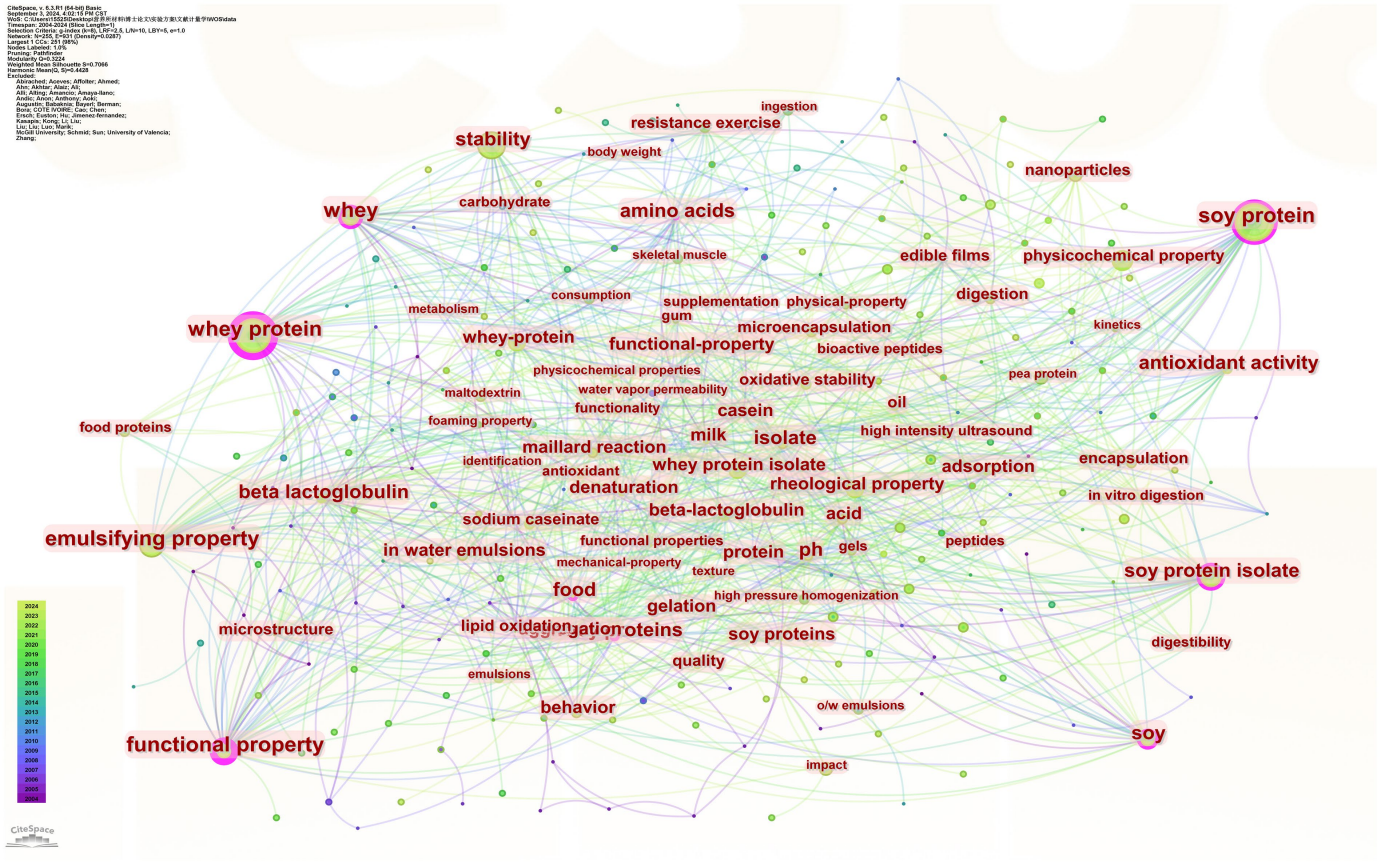
Keyword co-occurrence mapping.

Keywords are highly condensed and direct reflections of the research topic. The keyword co-occurrence analysis was performed using CiteSpace software (Figure 7). A total of 255 nodes were obtained with a network density of 0.0287 and the lines represent the links between the keywords

In order to present the main keywords as comprehensively as possible, CiteSpace software was used to obtain the top 30 keywords in terms of frequency, as shown in Table 2. Whey protein and soy protein were removed to include physicochemical properties and process properties, such as stability, emulsifying property, rheological property, and pH, as well as soy protein isolate and whey protein isolate, etc.

**Table 2:** Top 30 keywords in terms of frequency.

Number	Keywords	Count	Centrality
1	Soy protein	464	0.15
2	Whey protein	326	0.24
3	Stability	218	0.08
4	Whey	204	0.13
5	Emulsifying property	199	0.10
6	Soy protein isolate	175	0.10
7	Whey protein	162	0.08
8	Whey protein	158	0.15
9	Functional property	153	0.24
10	Isolate	151	0.08
11	Rheological property	132	0.03
12	pH	124	0.06
13	Soy	120	0.13
14	Physicochemical property	111	0.03
15	Whey protein isolate	109	0.03
16	Milk	107	0.07
17	Antioxidant activity	94	0.08
18	Beta-lactoglobulin	88	0.08
19	Behaviour	80	0.06
20	In water emulsions	73	0.03
21	Aggregation	72	0.04
22	Beta-lactoglobulin	70	0.06
23	Functional property	68	0.02
24	Food	68	0.12
25	Soy proteins	66	0.08
26	Oxidative stability	65	0.03
27	Encapsulation	63	0.05
28	Oil	53	0.04
29	Microstructure	50	0.03
30	Sodium caseinate	50	0.02

The controlled (co-)word analysis method uses the co-occurrence of words or noun phrases in the literature to determine the network formed by the keywords that we have retrieved from this literature set.

**Figure 8:**
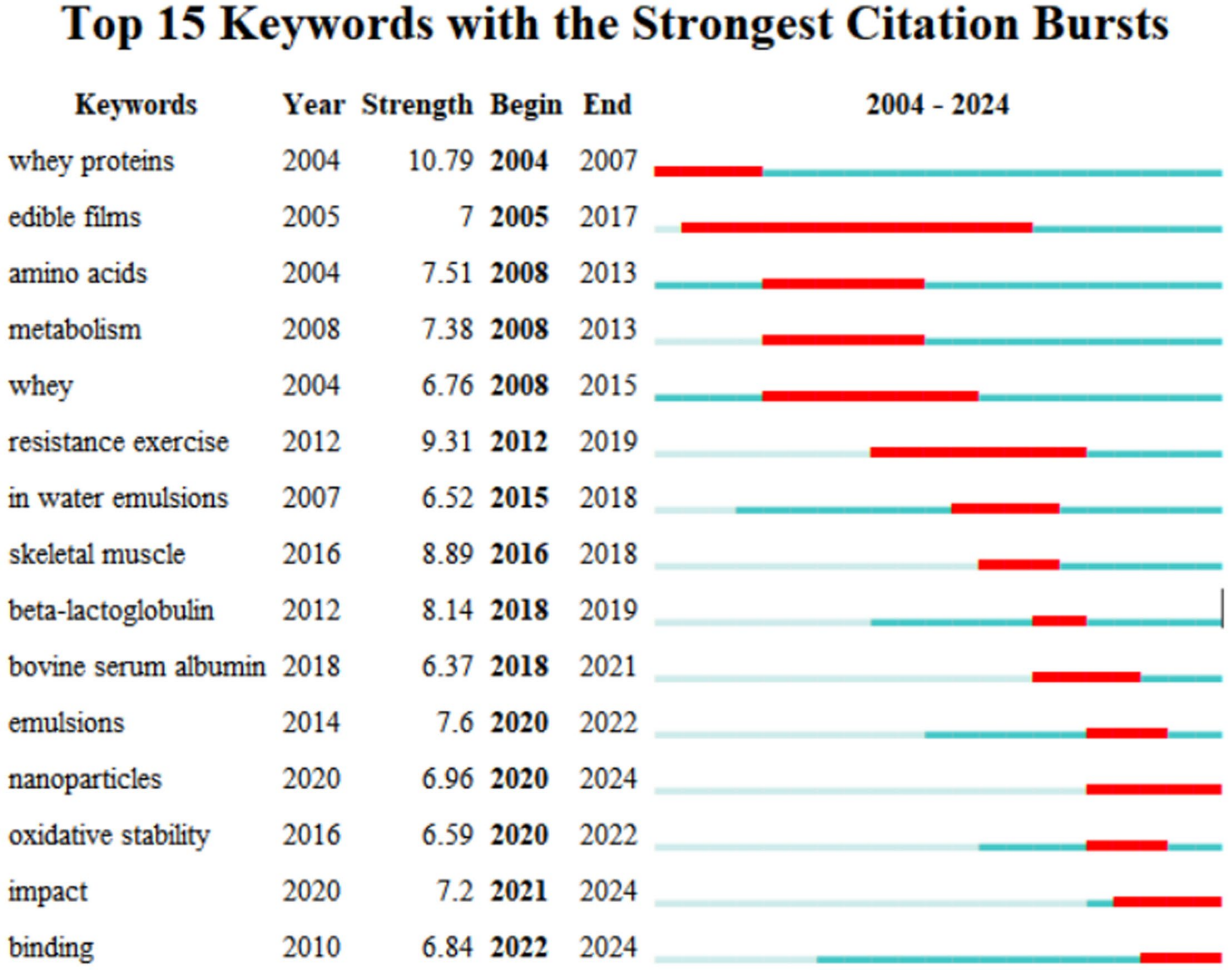
Top 15 keyword emergence mapping.

Emerging words reflected the increased intensity and frequency of certain keywords over a period of time, describing the research focus, hotspots, and evolutionary trends in the research field. It mainly reflected the research frontier and predicted the direction of research development (46, 47). Figure 8 shows the burst mapping of the top 15 keywords with the strongest burst intensity and analysing the bursts of soy and whey proteins, nanoparticles, and binding on the timeline lasting until 2024, which shows that the research will continue to be a hotspot for cutting-edge research in the coming years.

#### Keyword cluster analysis

The likelihood ratio (LLR) algorithm is used to produce a keyword clustering map, the more nodes and research hotspots are clustered. The smaller the cluster order is (44). Clustering modularity index Q-value and clustering contour index S-value are two assessment indexes for evaluating the clustering effect of the atlas. When the Q-value is greater than 0.3, it indicates that the division of clustering is significant; when the S-value is greater than 0.5, it indicates that the clustering is reasonable, and when it reaches 0.7, the clustering efficiency is high (20).

**Figure 9:**
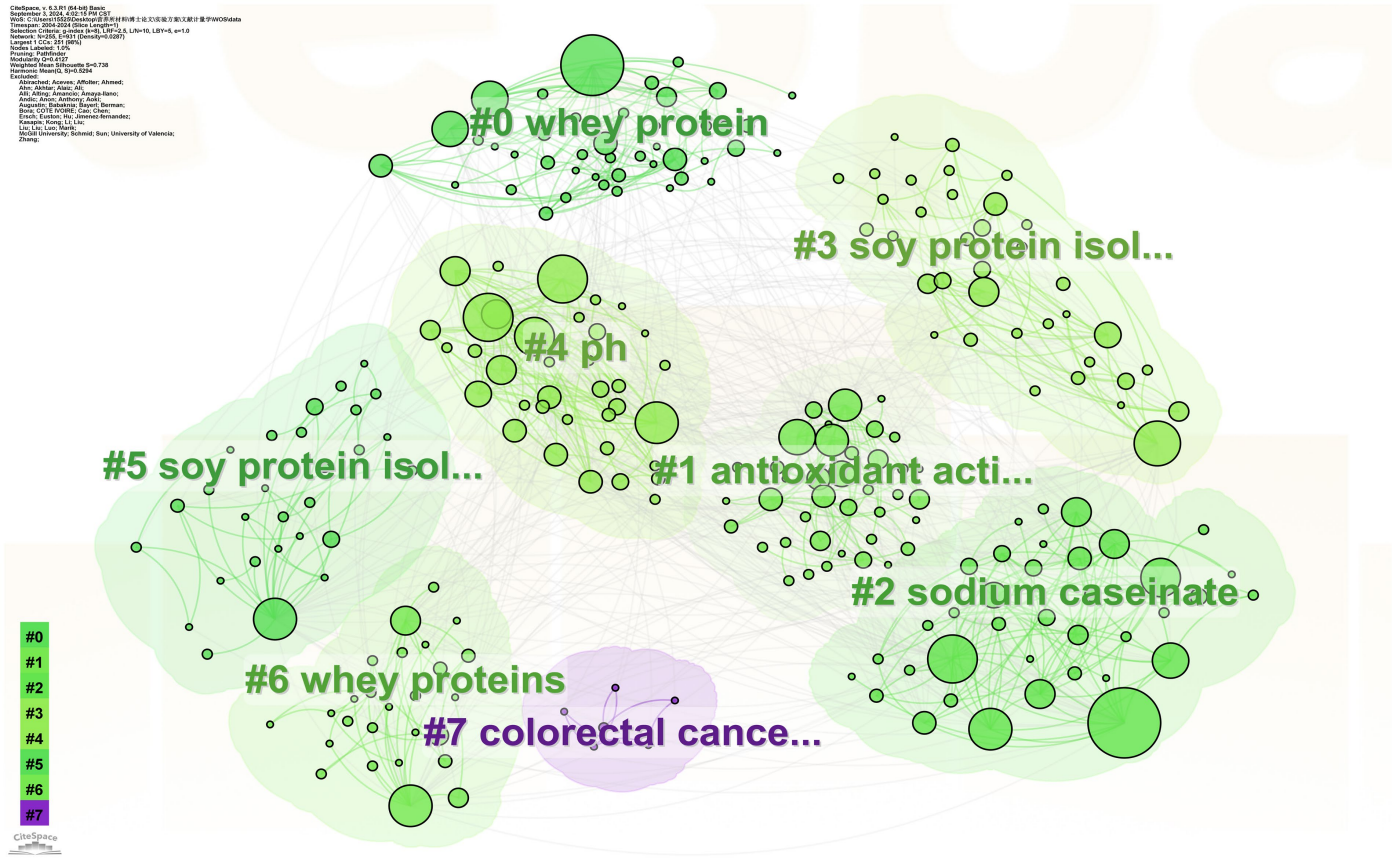
Keyword clustering network.

Keyword clustering can visualise the intrinsic connection between research topics. In this study, the Q-value of clustering was 0.4127 and the S-value was 0.738. The cluster analysis plot (Figure 9) formed a network of seven highly relevant and scalable keyword clusters, namely whey protein, antioxidant activity, sodium caseinate, soy protein isolate, pH, soy protein isolation, whey proteins, colorectal cancer, with contour values of 0.891, 0.493, 0.806, 0.702, 0.732, 0.772, 0.727, and 0.956, respectively. Excluding the keywords related to the topic, cluster #1 is antioxidant properties, and there are studies in the literature found that soy and whey proteins have antioxidant properties and can improve antioxidant indices in athletic and elderly populations, with soy proteins being more effective (48–50). Cluster #2 Sodium caseinate is a sodium salt of a protein found in cow’s milk and is often used as a biopolymer (51), emulsifier (52–56), and protein delivery vehicle in conjunction with soy protein and whey protein (57–59). Clustering #4 pH can directly affect protein content and properties (60). Cluster #7 is colorectal cancer, which can be inhibited to some extent by moderate amounts of soy protein and whey protein (61–63).

### Major research areas and their impact

**Figure 10:**
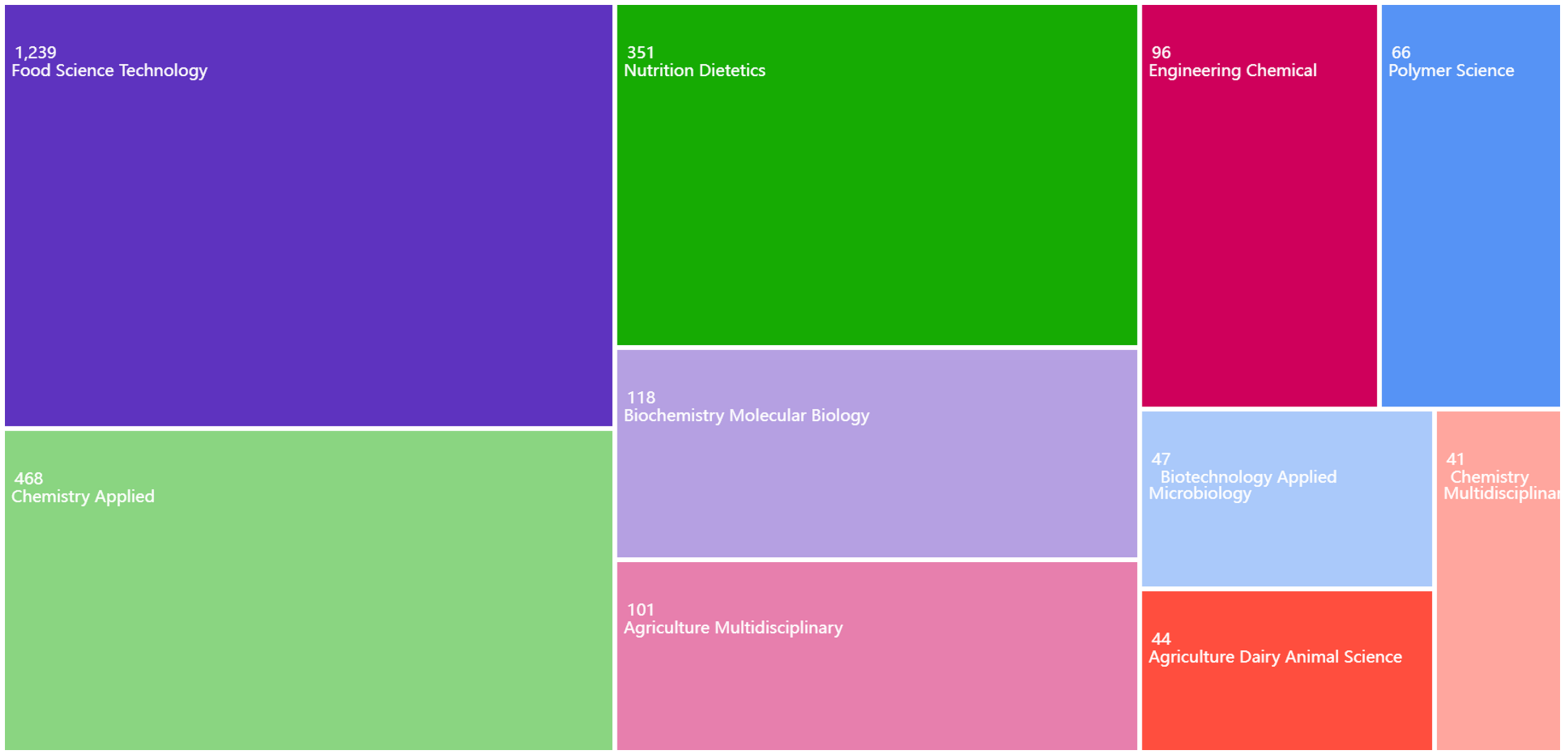
Soy protein/soybean protein and whey protein research discipline categories.

The self-guided search and analysis platform of the WOS database was used to count the disciplines in the field of soy protein/soybean protein and whey protein research and to draw a statistical map of the disciplines to which the relevant studies belonged (Figure 10).

The search was conducted from 1 January 2004 to 27 August 2024, and 1,888 articles were retrieved. Amongst them, 1,239 were in food science and technology, and 351 were attributed to nutrition. The relevant literature had focussed mainly on food science and technology, and these studies were gradually expanding into the discipline of nutrition as the nutritional and functional properties of proteins were studied in-depth. In recent years, the application of soy and whey proteins in nutritional supplementation, exercise recovery, and disease prevention interventions has received widespread attention as consumers focus on nutrition and the demand for healthier foods grows. Therefore, with increasing interdisciplinary research collaborations in a wide range of fields such as food science, nutrition, biochemistry, and molecular biology, these collaborations may contribute more to a comprehensive understanding of the functional properties of high-quality animal, plant, and microbial proteins and their health benefits. In addition, combined with the clustering network analysis of keywords, its research may gradually shift from the process of physicochemical properties to the direction of nutritional health, a shift that reflects the importance and in-depth exploration of the nutritional and health value of proteins.

## Discussion and conclusion

The General Office of the State Council of China has indicated that we should seek protein from plants, animals, and microorganisms, and also guide the consumption of nutritious and healthy food. Moreover, it has carried out the *National Nutrition Plan* in-depth, enhanced the nutrition and health standards system, and inspired enterprises to develop nutritious and healthy food products (64). Under its guidance, research on obtaining protein from plants, animals, and microorganisms has been continuously advancing. Amongst them, the dual protein, which is a mixture of soy protein and whey protein, has become a hot research area by virtue of its unique advantages. Specifically, researchers have found that the dual protein contains a variety of essential amino acids, which are nutritionally complementary; the types and ratios are more comprehensive and reasonable than those of single proteins; and its nutritional value is very similar to the amino acid standard model recommended by the FAO/WHO (Food and Agriculture Organization of the United Nations/World Health Organization) (65–67). In addition, the human digestibility of soy–whey protein blend can reach 96%, making it highly digestible and easily absorb by the human body (38). In recent years, with the rising demand for healthy nutrition and the development of food science and technology, research on dual protein has shown an increasing trend. On the one hand, in the research and development of dual-protein products, many researchers and enterprises have invested a lot of energy, aiming to develop more types of dual-protein products with better taste and adapted to the needs of different consumer groups, such as dual-protein compliant nutritional powder, dual-protein nutritional bars, dual-protein yoghurt, and so on (67–69). On the other hand, in terms of verifying the functional efficacy of dual protein, researchers have continued to explore the role of dual protein in promoting muscle growth, anti-fatigue, enhancement of immunity, lowering of blood lipids, improvement of nutritional status, and other aspects through a large number of experimental and clinical studies. These efforts have led to the gradual introduction of dual protein into the public eye, and its unique nutritional value has been accepted by the public. However, for the better and faster development of dual-protein research, it is necessary to understand the related knowledge. Therefore, bibliometrics was used to quickly grasp the research dynamics in this field and to deeply analyse the research hotspots in related literature (69–73).

In this study, an in-depth analysis of the literature available on soy protein/soybean protein and whey protein from 2004 to 2024 was conducted using knowledge mapping. Specifically, the study included 1,847 documents with the aim of mining the literature for data and potential information. The trends in publication, the authors, the cooperation between institutions, the research hotspots, and the discipline development trends are considered comprehensively. At present, the studies on soy protein and whey protein focussed on structure, functional properties, and nutrition. As far as the research results were concerned, China’s research in this field had a significant global impact and made the most outstanding contribution, which will be a guide for the continual expansion of practical applications and research on industrial development trends in the related fields in the future. In terms of the institutions issuing papers in the relevant literature, the results of the analyses showed that although deeper clusters of cooperation have not yet been formed, there were already a number of research institutions that have demonstrated significant activity within the relevant research field. These institutions may have established a certain degree of influence and recognition within the research field by publishing multiple papers and participating in international collaborative projects. In addition, although the collaborative network was not yet fully mature, this initial level of activity and participation laid the foundation for broader collaborations in the future and helped to promote further development and innovation in the field of soybean protein and whey protein studies.

In terms of research contributions, China dominates the field of soy protein and whey protein research, with a much higher number of publications than other countries. However, there are differences in specific research focus areas compared to other leading countries. In terms of sustainability, for example, countries such as the Netherlands have been very productive in driving a shift to a more plant-based diet, with research focussing on how plant proteins (such as soy proteins) can safeguard the nutritional needs of the human body whilst reducing environmental impacts.

From the perspective of solving practical problems, interdisciplinary cooperation can integrate the advantageous resources of all parties. For example, when researching the role of soy protein and whey protein in disease prevention and treatment, food science researchers can develop appropriate protein product dosage forms, nutritionists can assess the effects through human trials, biochemists and molecular biologists can investigate the mechanism of action, and multidisciplinary teamwork can efficiently overcome the problems and vigorously promote the progress of research. In addition, interdisciplinary cooperation can stimulate innovation and vitality, and the collision of different disciplines can often give rise to new research ideas and methods, opening up new directions for protein research.

This paper provided a systematic and comprehensive analysis of the research areas and development trends of soy and whey proteins through bibliometric and visual analyses, providing some references for researchers. However, there are some limitations to this paper: (1) The dataset covered a small range of topics, resulting in analysis results that may not be comprehensive; (2) Some excellent and high-quality literature may not be highlighted due to low citation rates, leading to limitations in the results; (3) There was a certain delay in updating the database, and the latest published literature may not be included in time, thus affecting the timeliness of the analysis.

In order to reduce these limitations in future bibliometric analyses, the following approach could be taken to: (1) expand the thematic scope of the dataset: Integrate multiple databases of diverse types and fields to access a broader spectrum of literature resources. According to the research objectives, reasonably broaden the thematic scope by incorporating relevant literature from fringe areas and interdisciplinary fields, thereby preventing the omission of crucial information due to a narrowly defined theme; (2) improve the literature evaluation system: establish an expert evaluation mechanism, invite experts in the field to evaluate the literature qualitatively, identify high-quality literature that has potential value despite low citation rates, and pay attention to the quality of the literature’s content, innovativeness, scientificity of the research methodology and other intrinsic characteristics; and (3) establish a real-time monitoring and updating mechanism. Leverage web crawler technologies to regularly scour academic resource websites. By doing so, newly published literature can be promptly detected and incorporated into the database.

From the analysis of the results of this study, the following recommendations are given: (1) Further develop efficient and environmentally friendly extraction methods and improve the functionality and nutritional value of proteins through modification techniques; (2) increase the research and development of functional foods, such as the development of protein foods with specific functions; (3) explore the nutritional efficacy and mechanism of mixed protein (or dual protein) of soy protein and whey protein, and lay the theoretical foundation for the development of dual protein specialty foods; (4) strengthen international cooperation and exchange, share research resources, and promote the balanced development of protein research worldwide.

For researchers, institutions, and policymakers, the following recommendations are made: (1) Researchers focus on foreword and innovation research to analyse the relationship between protein structure and function at the molecular level and develop protein products with unique functions; (2) Research institutions rationally adjust the allocation of research resources, provide a platform for cooperation and resource support for researchers from different disciplines, and acquire advanced experimental equipment to improve the overall research strength of research institutions; (3) Policymakers set up special funds for interdisciplinary research, establish a diversified evaluation mechanism, and encourage enterprises and research institutions to increase their investment in protein research and improve research efficiency.

With socio-economic development and increased awareness of nutritional health, research organisations are likely to form closer and more effective research networks through enhanced collaboration and the sharing of resources and knowledge to address more complex research challenges and issues.

## Data Availability

The original contributions presented in the study are included in the article/supplementary material, further inquiries can be directed to the corresponding author.
